# Relationship of Binge Drinking with Impairments Affecting Memory and Executive Function among University Students: A Cross-Sectional Study in Northern Spain

**DOI:** 10.3390/ijerph182111508

**Published:** 2021-11-01

**Authors:** Manuel Herrero-Montes, Cristina Alonso-Blanco, María Paz-Zulueta, Amada Pellico-López, Laura Ruiz-Azcona, Carmen Sarabia-Cobo, Ester Boixadera-Planas, Paula Parás-Bravo

**Affiliations:** 1 Department of Nursing, Faculty of Nursing, University of Cantabria, 39008 Cantabria, Spain; manuel.herrero@unican.es (M.H.-M.); amada.pellico@scsalud.es (A.P.-L.); laura.ruiz@unican.es (L.R.-A.); carmen.sarabia@unican.es (C.S.-C.); paula.paras@unican.es (P.P.-B.); 2 IDIVAL, Research Nursing Group, 39008 Cantabria, Spain; 3 Department of Physiotherapy, Occupational Therapy, Rehabilitation, and Physical Medicine, University Rey Juan Carlos, 28922 Madrid, Spain; cristina.alonso@urjc.es; 4 IDIVAL, GI Derecho Sanitario y Bioética, GRIDES, 39008 Cantabria, Spain; 5 Cantabria Health Service, Avda. Derechos de la Infancia, 31, 39340 Cantabria, Spain; 6 Servei d’Estadística Aplicada of the UAB, Autonomous University of Barcelona, 08193 Barcelona, Spain; ester.boixadera@uab.cat

**Keywords:** binge drinking, alcohol-related disorders, young adulthood, alcohol drinking in college, brain function, psychological test

## Abstract

Binge drinking (BD) is a common practice among college students. Alcohol consumption may affect brain structures that continue to develop in early adulthood. The aim of this study was to analyze the relationship of BD with impairments affecting memory and executive function among university students. A cross-sectional study was conducted among students (aged 18–30 years) enrolled for the academic year 2018–2019 at the Faculty of Nursing of the University of Cantabria (Spain). Data collection included sociodemographic and academic information, together with alcohol and drug use information, collected by means of a semi-structured questionnaire. A battery of validated tests was used to gather neuropsychological variables. A total of 142 participants were included, of which 88.03% were women. Up to 38.03% were classified as BD. No differences were found in memory tests. Regarding executive function, better performance in the Stroop Color and Word Test was observed in non-BD but the results were not statistically significant. In conclusion, no relationship was found between memory and executive function and BD, although better performance in executive function, specifically inhibitory control, was observed in non-BD.

## 1. Introduction

Alcohol is the most widely consumed psychoactive substance in many countries. In data from 2018, around 43% of the world’s population and over 50% of Europeans had consumed alcohol in the past 12 months [1]. In Spain, in 2017, approximately 90% of the population over 14 years of age had consumed alcohol in their lifetime and approximately 75.2% had done so in the last year [2]. Alcohol consumption is a frequent habit among young adults [3], considering that worldwide, over a quarter of young people aged 15–19 years old consume alcohol, with the highest prevalence rate found in the WHO European Region. In Spain, during the period of 2010–2017, around 60% of young people aged 15–34 years had consumed alcohol in the last 30 days [2].

Among young people, there is a predominance of alcohol consumption characterized by a high intake in a short period of time, followed by periods of abstinence, known as binge drinking (BD) [4]. The definition of BD does not have a consensus in the scientific literature. The National Institute on Alcohol Abuse and Alcoholism published, in 2004, one of the most widespread definitions internationally: “pattern of drinking alcohol that brings blood alcohol concentration to 0.08 percent—or 0.08 g of alcohol per decilitre—or higher. For a typical adult, this pattern corresponds to consuming 5 or more drinks (male), or 4 or more drinks (female), in about 2 h” [5]. This definition considers quantity and period of consumption, but lacks the variable frequency of consumption, which is important for determining the health and social consequences of this pattern. In Spain in 2011, Parada et al. [6], in an attempt to collect all the parameters that have been shown to be relevant in the definition and adapt it to the national reality, proposed that: “BD is the consumption of six or more alcoholic drinks for men (60 g)-5 or more for women (50 g)-on a single occasion (within a two-hour period) at least once in the past 30 days”.

Moreover, BD is a pattern of consumption that takes place worldwide, with a peak prevalence between the ages of 20–24. In 2016, the prevalence in adolescents over 15 years of age was 18.2%, with the highest rates found in the European region at 26.4% [1]. Moreover, men have a higher prevalence than women [2,7]. In Spain, the prevalence of BD in the general population is around 15%, with particularly high figures among young people, with a prevalence of approximately 30% in men between 20–29 years of age and 17–20% among women of the same age [2], and up to 32.3% in students between 14–18 years of age [8].

Among young university students, alcohol consumption and the practice of BD are frequent habits [9,10,11], with higher prevalence rates than the general population of the same age [12], over 40% in the last 30 days [9,10,13].

The cerebral cortex, the limbic system, and the cerebellum are structures vulnerable to the effects of alcohol consumption. Moreover, these brain regions, such as the cortico-limbic regions, continue to develop throughout adolescence and early adulthood. A systemic review shows than delayed development of key frontal executive-control regions may predispose youth to binge drink [14]. A literature review found that adolescents with alcohol use disorders often show poorer neurocognitive performance, alterations in gray and white matter brain structure, and discrepant functional brain activation [15]. Moreover, alcohol use during adolescence is associated with prefrontal volume abnormalities, including white matter differences [16].

Some studies associate excessive alcohol consumption or BD with poorer verbal declarative memory [17,18]. However, few studies have been aimed at evaluating the relationship between BD patterns of alcohol consumption and executive function. Salas et al. [10] found that BD in college students was associated with a lower performance in some executive functions tests. In young people, García-Moreno et al. [19] also found that BD is associated with poorer performance in neuropsychological tasks which depend on correct prefrontal cortex functioning. A systematic review shows than BD is associated with deficits in some types of memory like verbal memory and some executive functions, principally poor inhibitory control [20] but the results obtained are not conclusive, and it is necessary to continue researching in this area.

The aim of this study was to analyze the relationship of BD with impairments affecting memory and executive function among university students.

## 2. Materials and Methods

### 2.1. Study Design

A cross-sectional study was performed. The study design was approved by the Ethics Committee of Cantabria, Spain (Code: 2015.102). All procedures were conducted according to the Declaration of Helsinki [21] and participants read and signed a written consent form prior to their participation in the study. The data were anonymized and treated confidentially in accordance with the Personal Data Protection legislation [22].

### 2.2. Participants

**Figure 1:**
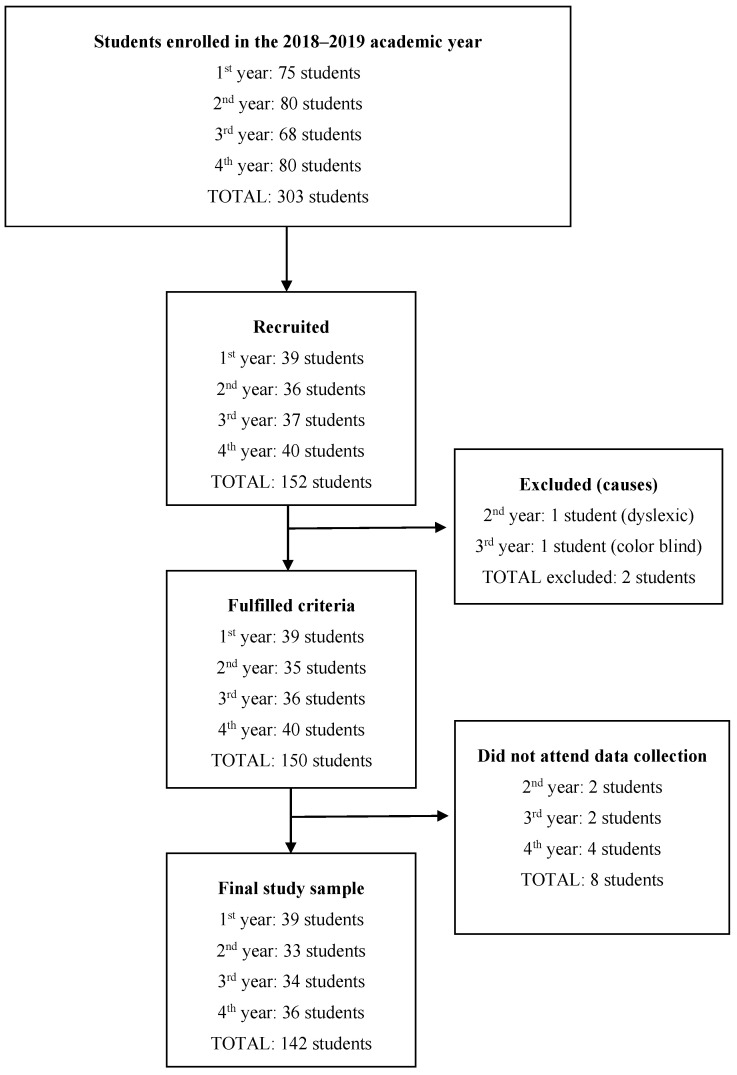
Diagram of study participation.

All university students enrolled in the 2018–2019 academic year at the Faculty of Nursing of the University of Cantabria (Spain) aged between 18 and 30 years were included in the study. Those with sensory deficits or diseases that prevented the performance of the neuropsychological tests were excluded. A total of 303 students initially enrolled in this study, of which 142 finally participated (Figure 1).

### 2.3. Variables and Measuring Tools

#### 2.3.1. Sociodemographic and Academic Data. Alcohol, and Other Drug Consumption

A semi-structured interview was conducted in which sociodemographic and academic variables were collected (gender, age, place of residence, mother’s and father’s level of studies, and mean grade on the academic transcript.

Data were collected on quantity and pattern of alcohol consumption at different periods: some time in life, during the last 12 months and during the last 30 days. The standard drink (SD) was used to measure the amount of alcohol consumption. In Spain, a SD corresponds to a drink containing 10 g of alcohol. A table was provided showing the alcohol content in SD of the most commonly consumed alcoholic beverages in Spain.

Alcohol consumption at some point in life was collected as a dichotomous variable (Yes/No) and the age of first alcohol consumption was also asked.

During the last 12 months: the SD consumed on a typical drinking day; the frequency of drinking (every day, 5–6 times a week, 3–4 times a week, 2 times a week, once a week, 2–3 times a month, once a month, 3–11 times in the last year, 1–2 times in the last year or no drinking, 1–2 times in the past year or never) and the frequency of drinking 6 SD (men) or 5 SD (women) or more of alcoholic beverages (every day, 5–6 times a week, 3–4 times a week, 2 times a week, once a week, 2–3 times a month, once a month, 3–11 times in the past year, 1–2 times in the past year or never) were measured.

“During the last 30 days: number of times you have been drunk and maximum alcohol intake in a 2-h period”. The question on maximum alcohol intake in a 2-h period in the last 30 days was used to classify students as BD or non-BD according to the definition proposed by Parada et al. [6]: “consumption of six or more alcoholic drinks for men (60 g)-5 or more for women (50 g) on a single occasion (within a two-hour period) at least once in the past 30 days”.

The questionnaire included the Alcohol Use Disorders Identification Test (AUDIT) developed by the WHO [23] which has been validated in Spain by Rubio et al. [24] and presenting adequate psychometric properties for the detection of alcohol problems in college students [25], as well as for the distinction between BD and non-BD consumption in this population group [26]. The cut-off points for detecting risk drinkers in the university population in Spain are set at eight points for men and six points for women [25]. In addition, data on tobacco, cannabis or cocaine use in the last year was collected by asking: “During the last 12 months, how often have you used tobacco/cannabis/cocaine?”.

#### 2.3.2. Neuropsychological Variables

##### Memory

The Wechsler Memory Scale-Third Edition (WMS-III) Logical Memory I Test [27] was used which evaluates immediate episodic verbal memory and the Rey-Osterrieth Complex Figure Test (ROCFT) [28] was used for visual memory [28,29].

The administration of the WMS-III Logical Memory I Test consists of the examiner reading two stories and immediately after reading each of the texts the subject is asked to recall what they remember from each of them. The second story is read twice. The total recall score is the sum of each of the scores of the text units: A, B (first recall) and B (2nd recall). The score is between 0 and 75.

The ROCFT consists of copying and, after a given period, reproducing a complex geometric figure. When administering this test, first, the subject is shown the picture and asked to reproduce it while the figure is placed in front of them (Copy); then, after 3 min, the person is asked to reproduce the figure from memory (Delayed Recall), following the instructions of the manual in Spanish [28].

For the evaluation of the process of copying and recalling, the figure is broken down into 18 elements and the same score is awarded. Thus, the direct score obtained is between 0 and 36 points in each phase.

##### Executive Functions

The Stroop Color and Word Test (SCWT) [30] was used to measure inhibitory control, the Wechsler Adult Intelligence Scale-III (WAIS-III) Digit span [31] was used to measure working memory and the Trail Making Test (TMT) [32] was used to measure cognitive flexibility.

The SCWT consists of three A4 pages containing five columns with 20 elements each. The first page features the words “RED”, “GREEN” and “BLUE” in black and in a random order, with none of the words appearing twice in a row. The second has 100 of the same elements (“XXXX”) printed in blue, green, or red font. As on the first page, no color is repeated twice in a row. The third is a combination of the words of the first with the colors of the second, i.e., the first written word is the same as on the first sheet, although printed in the color of the first “XXXX” of the second sheet. The color of the ink never matches the meaning of the word.

The administration is performed in the same order, as follows (each for 45 s duration):

- First sheet: read the text of each word.
- Second sheet: say the color of each XXXX.
- Third sheet: say the color of each word.

The number of items named on each of the sheets is recorded (errors are not counted, but represent a worse score in the execution of the test since the item must be repeated correctly) obtaining three scores: words reading score of the first sheet; color naming score of the second sheet and color-color interference score of the third sheet. Subsequently, the predicted color-word score (SCWT-PC’) is calculated, which derives from the idea that the easiest way to complete the items on the third sheet is to read the word and then name the color. The interference score (SCWT-INTERF) is the difference between SCWT-PC and SCWT-PC’.

The WAIS-III Digit Span Test consists of two exercises that are administered independently: digit span forward and digit span backward. In both, the examinee is asked to repeat a series of digits read by the examiner, in the first exercise these are repeated in the same order (digit span forward) and in the second exercise these are repeated in the reverse order (digit Span Backwards). The test consists of eight items for digit span forward and seven for Digit Span Backwards. Each of the items is composed of two sets of numbers of the same length. Two attempts were administered for each item regardless of whether the response to the first was correct [31]. The score is equal to the number of digits of the longest error-free series, between 0 and 9 in direct digits and between 0 and 8 in inverse digits, following the methodology of the NEURONORMA study [33].

The TMT consists of two parts: part A consists of joining numbers from 1 to 25, randomly distributed on a sheet of paper, in order, from smallest to largest; part B consists of joining numbers from 1 to 13, interspersing them with letters in alphabetical order. The test was administered according to the procedure proposed by Reitan [32], quantifying the time taken to complete each part.

### 2.4. Procedure

Participants were recruited via informative sessions and posters placed in the faculty. Two researchers conducted the data collection. Both were trained by a psychologist, who was a member of the research team, and who taught how to administer the psychological tests which were carried out in pencil and paper. Two independent offices with good conditions were available for this task. Data collection was carried out between December 2018 and January 2020. The data collection required approximately 30 min for each participant. Participants were required not to consume alcohol or any other drug on the day of the assessment.

The data from the first 30 participants were used as a pilot study, and after their analysis the research team met without making substantial changes to either the questionnaires or the administration procedure. These data were also included later in the total sample.

### 2.5. Statistical Analysis

For the statistical analysis, a distinction was made between categorical and quantitative variables. A descriptive bivariate analysis of BD and the remaining study variables was performed. Categorical variables are presented as counts and percentages of the total and of the BD and non-BD groups. These descriptive results were completed with the Pearson’s Chi-Square test or the Likelihood Ratio Chi-Square test as appropriate, to contrast the independence between each variable and BD. Quantitative variables are presented with basic descriptive statistics (n, mean, median, SD and quartiles) for the total and for the BD and non-BD groups. These results are complemented with the Wilcoxon rank-sum test to contrast the distributions of the quantitative variable between non-BD and BD. In cases of normal distribution of the variable in each non-BD and BD group, the results of the *t*-test to compare means of independent groups are also presented: non-BD and BD.

The statistical analysis was performed using SAS v9.4 software, SAS Institute Inc., Cary, NC, USA. Statistical decisions were made using 0.05 as the level of significance.

## 3. Results

In total, 142 participants were included in the study. Eighty-eight participants belonged to the non-BD group and 54 to the BD group, corresponding to 61.97% and 38.03% respectively of the total sample. Up to 11 participants (7.75% of the total) were teetotalers, meaning that they had not consumed alcohol during the last year, and six of these had never consumed alcohol (4.22% of the total).

### 3.1. Sociodemographic, Academic, Alcohol and Other Drug Use Variables

The complete data can be seen in Table 1 and Table 2. Women constituted 88.03% of the participants. A higher proportion of women was observed in the non-BD group: 92.05% women and 7.95% men, whereas in the BD group there were 81.48% women and 18.52% men, although this difference was not statistically significant (*p* = 0.0598).

Regarding the place of residence, 83.33% of the BD group lived in the family home compared to 77.27% of the non-BD group (*p* = 0.3845).

The mother’s level of education was higher in most categories in the non-BD participants, for example, in non-BD, 33 participants (37.5%) indicated that their mother had a university education, compared to only 10 participants (18.52%) who were BD, although no statistically significant differences were detected (*p* = 0.0631).

The median age of onset of alcohol use in non-BDs was 16.00 (Q1 = 15, Q3 = 17) years and 15.00 (Q1 = 14, Q3 = 15) years in BDs (*p* < 0.0001); six participants have not been included in this contrast because they have never drunk alcohol. The median total direct score of the AUDIT questionnaire in non-BDs was 2 (Q1 = 1, Q3 = 4) points and 7 (Q1 = 5, Q3 = 12) points in BDs (*p* < 0.0001). 3 participants (14.77%) non-BD and 36 participants (66.67%) BD are risk drinkers (AUDIT total >= 8 in men or >= 6 in women) (*p* < 0.0001). Regarding the frequency of drinking over the last 12 months, 39 non-BD participants (44.32%) consumed alcoholic beverages “less than once a month” and 38 (43.18%) did so “1 to 3 times a month” vs. 21 BD participants (38.89%) who did so “two or more times a week” and 19 (35.19%) who drank alcohol “1 to 3 times a month” (*p* < 0.0001).

Seventy-one non-BD participants (80.68%) indicated that they had never smoked, 15 people (17.05%) indicated that they smoked “Some days” and two participants (2.27%) indicated smoking “Every day”. Conversely, in the group of BD participants, 28 (51.85%) indicated “Never” smoking, 17 students (31.48%) indicated smoking “Some days”, and nine participants (16.67%) reported smoking “Every day” (*p* = 0.0003).

Regarding cannabis use, 79 non-BD participants (89.77%) reported that they had never used cannabis in the past year and nine participants (10.23%) used it on “Some days”. 31 “BD” participants (51.41%) did not use cannabis and 23 (42.59%) used it “Some days” (*p* < 0.0001). None of the participants reported having used cocaine in the past year.

### 3.2. Memory and Executive Functions

The results of the neuropsychological tests that measure memory and executive functions are displayed in Table 3.

As for the two tests used to measure memory, the median of the WMS-III Logical Memory I Test in non-BDs was 50 (Q1 = 45, Q3 = 55) points and 50.5 (Q1 = 47, Q3 = 53) points in BDs.

The median ROCFT delayed recall test phase score in non-BDs was 29 (Q1 = 22, Q3 = 32) points and 29 (Q1 = 25, Q3 = 30) points in BDs.

No statistically significant differences were found for the WMS-III Logical Memory I Test or the ROCFT delayed recall test.

Regarding executive functions, in the WAIS-III Digit Span test, digit span forward, digit span backwards and digit span total scores were analyzed separately, with no statistically significant differences. In the SCWT direct scores, a better performance in the SCWT-W subtest was observed in the non-BD group, 51.52 (SD 9.02) points versus 50.26 (SD 7.85) in the BD group. The difference was not statistically significant (*p* = 0.3965). Higher mean SCWT-INTERF scores were also observed in the non-BDs, 5.06 (SD 6.20), versus the BDs, 3.71 (SD 6.52). Likewise, the difference was not statistically significant (*p* = 0.2184). No statistically significant differences were found in the TMT.

## 4. Discussion

No relationship between BD with impairments affecting memory and executive function among university students was found in our sample.

We failed to find a relation between verbal memory and BD. A systematic review by Carbia et al. [20] claims that the pattern of BD in college students is associated with difficulties in verbal memory although the authors only found eight studies that evaluated this variable, using different tests based on learning lists or story recall.

Other studies conducted in Spain with university students who, like ours, also use the logical memory subtest of the WMS III generally showed worse performance in BD students than non-BD students [13,34,35]: Parada et al. [34], in a cross-sectional study with 122 participants, students with BD drinking patterns demonstrated significantly lower recall of verbal material than non-BD students; Mota et al. [35], in a longitudinal study with 89 young people reported that continued BD drinkers recalled less information than non-BD and ex-BD drinkers; and Carbia et al. [13] in a prospective study with a sample of 155 participants and a six-year follow-up, found that a stable pattern of BD alcohol consumption was associated with difficulties in remembering stories. Differences with our results could be explained by the definition of BD used in these studies, which generally increase the threshold of consumption to consider a subject as BD; the study populations, among which some are students and others are young people in general; or the methodology used, i.e., two cross-sectional studies and one longitudinal.

We also found no differences in visual memory between the two groups of young people in accordance with the results of the review by Carbia et al. published in 2018 [20], including 20 studies analyzing visual memory and BD, five of which used the ROCFT reproduction test as used in our study. Salas et al. [10] also found no differences in a population with similar characteristics, i.e., 206 undergraduate physical therapy students in Spain, using the ROCFT reproduction test for measurement.

Understanding that executive function encompasses working memory, inhibitory control, and cognitive flexibility, we also found no differences for the performance on neuropsychological tests measuring these functions between nursing students with a BD and non-BD drinking pattern.

Working memory, understood as the capacity to maintain and reorder information and measured with the WAIS-III inverse digits test, is not affected by BD practice, and we also found no differences in direct digits of the same test, which is considered to measure attention [36] and is mainly related to the maintenance of learned information. Carbia et al. [20] in their systematic review included 16 studies that analyzed working memory with different instruments and similarly found no associations between BD and attention or working memory problems measured with the digit test, although it seems that BD could have some difficulty in performing other tests, which also measure working memory and require the performance of more complex information manipulation tasks.

In terms of inhibitory control, which refers to the ability to suppress predominant, inappropriate, or impulsive responses and is measured with the SCWT test, no differences were observed between BD and non-BD participants, although non-BD participants obtained better test scores. Carbia et al. [18] in their systematic review, included eleven studies measuring this variable, with nine different instruments, and found that, in general, it seems that BD in youth is associated with lower inhibitory control, although the heterogeneity of the measurement instruments makes it difficult to compare results. Garcia-Moreno et al. [19] used the SCWT, as in our study, to compare inhibitory control between young BD, young non-BD drinkers and abstainers and found better performances in abstainers than in BD, although we included non-BDs and abstainers in the same group; therefore, it is to be expected that the differences with BD are smaller. Salas et al. [10], in a sample similar to ours, students enrolled in the Physical therapy Degree, also found no significant differences in the performance of the SCWT test.

According to our findings, cognitive flexibility, understood as the ability to switch from one thought to another or to alternate between tasks, is not altered by BD. Carbia et al. [20] in their systematic review, which includes eight studies measuring this function with seven different instruments, also found no clear evidence of a relationship, but there is evidence of worse cognitive flexibility in BD, although, again, the difference in the tests used in each study makes it difficult to compare results. Conversely, Salas et al. [10] in their study among physical therapy students and using the TMT, did find an association between BD and test results, although differences in the characteristics of the sample, such as cannabis use, gender distribution or age, could influence the results.

This study has been carried out on nursing students, which could be a limitation due to the specificity of the sample, but it provides data from a very specific population that has not been frequently studied. In addition, few papers use a set of neuropsychological tests such as ours, which explores numerous aspects of memory and executive function.

The main limitation of the study is probably the lack of consensus in the scientific community on the definition of BD and the absence of a validated instrument to identify and measure this pattern of consumption. To address this limitation, we have used a definition that includes the aspects that are usually included in other definitions and that is adapted to the circumstances of our context. In addition, the fact that this is a cross-sectional study and that the mean age of the participants is low probably makes it difficult to detect neuropsychological alterations.

Another limitation of the study is that the sample is small and heterogeneous in many variables, and is composed exclusively of nursing students, mostly women. This could be the reason for not finding significant differences in the variables studied and it conditions the extrapolation of the results to both sexes and makes it difficult to study the differences in the variables between men and women.

Further longitudinal studies are needed to research this subject and confirm these findings, and to explore whether BD-type consumption in youth has repercussions on neurocognitive performance in memory or executive function tests in adulthood.

## 5. Conclusions

Performance in neuropsychological tests measuring verbal and visual memory and neuropsychological tests measuring executive function were not related to BD alcohol consumption patterns in our sample. Nonetheless, better performance was found in some of the tests measuring executive function, specifically inhibitory control, in non-BD subjects. Inhibitory control is of special interest in relation to BD, since low levels of inhibitory control could lead to an increase in BD-type consumption, and therefore future studies are needed to investigate this relationship.

## Figures and Tables

**Table 1:** Sociodemographic and academic data.

	Total (n = 142)				Binge Drinkers							
					No (n = 88)				Yes (n = 54)			
	n		%		n		%		n		%	
**Gender**												
Female	125		88.03%		81		92.05%		44		81.48%	
Male	17		11.97%		7		7.95%		10		18.52%	
					*p* = 0.0598 ^b^							
	Mean	SD	Range	Median	Mean	SD	Range	Median	Mean	SD	Range	Median
**Age**	20.73	2.67	18–30	20.00	20.77	2.77	18–30	20.00	20.67	2.53	18–28	20.00
					*p* = 0.9830 ^a^							
**Mean grade**	7.18	0.9	4.55–9.11	7.3	7.24	0.96	4.55–9.11	7.44	7.07	0.79	4.91–8.65	7.27
					*p* = 0.1753 ^a^							
	n		%		n		%		n		%	
**Place of residence**												
Family home	113		79.58%		68		77.27%		45		83.33%	
Noy in the family home	29		20.42%		20		22.73%		9		16.67%	
					*p* = 0.3845 ^b^							
**Maternal level of studies**												
University	43		30.28%		33		37.5%		10		18.52%	
Secondary/vocational training	57		40.14%		32		36.36%		25		46.3%	
Primary	36		25.35%		21		23.86%		15		27.78%	
No studies	6		4.23%		2		2.27%		4		7.41%	
					*p* = 0.0631 ^c^							
**Paternal level of studies**												
University	45		31.69%		27		30.68%		18		33.33%	
Secondary/vocational training	55		38.73%		36		40.91%		19		35.19%	
Primary	36		25.35%		22		25,00%		14		25.93%	
No studies	6		4.23%		3		3.41%		3		5.56%	
					*p* = 0.8671 ^c^							

Wilcoxon Test (^a^); Chi-Square Test (^b^); LR-Chi-Square Test (^c^); SD (standard deviation).

**Table 2:** Consumption of alcohol and other drugs.

	Total (n = 142)				Binge Drinkers							
					No (n = 88)				Yes (n = 54)			
	Mean	SD	Range	Median	Mean	SD	Range	Median	Mean	SD	Range	Median
**Age of onset of alcohol use (n = 136)**	15.24	1.65	8–19	15.00	15.67	1.66	8–19	16.00	14.59	1.43	12–18	15.00
					*p* =< 0.0001 ^a^							
**AUDIT total**	4.96	4.55	0–20	3	2.94	2.92	0–14	2	8.26	4.83	1–20	7
					*p* < 0.0001 ^a^							
**Substance Use in the Last 12 Months**												
	n		%		n		%		n		%	
**Alcohol**												
Less than monthly	41		28.87%		39		44.32%		2		3.7%	
1 to 3 times a month	57		40.14%		38		43.18%		19		35.19%	
Once a week	17		11.97%		5		5.68%		12		22.22%	
2 or more times per week	27		19.01%		6		6.82%		21		38.89%	
					*p* < 0.0001 ^b^							
**Tobacco**												
Never	99		69.72%		71		80.68%		28		51.85%	
Some days	32		22.54%		15		17.05%		17		31.48%	
Every day	11		7.75%		2		2.27%		9		16.67%	
					*p* = 0.0003 ^b^							
**Cannabis**												
Never	110		77.46%		79		89.77%		31		57.41%	
Some days	32		22.54%		9		10.23%		23		42.59%	
					*p* < 0.0001 ^b^							
**Cocaine**												
Never	142		100.00%		88		100.00%		54		100.00%	

Wilcoxon Test (^a^); Chi-Square Test (^b^); SD (standard deviation); AUDIT (Alcohol Use Disorders Identification Test).

**Table 3:** Memory and executive functions.

	Total (n = 142)				Binge Drinkers							
					No (n = 88)				Yes (n = 54)			
	Memory											
	Mean	SD	Range	Median	Mean	SD	Range	Median	Mean	SD	Range	Median
**WMS-III** **Logical memory I**	37.07	8.32	13–60	38	36.98	8.29	13–53	38	37.22	8.45	21–60	38
					*p* = 0.8200 ^a^							
**ROCF** **Delayed Recall**	26.82	6.05	8–36	29	26.33	6.65	8–36	29	27.63	4.88	14–36	29
					*p* = 0.5720 ^a^							
	**Executive Functions**											
	Mean	SD	Range	Median	Mean	SD	Range	Median	Mean	SD	Range	Median
**WAIS-III**												
**Digit Span Forward**	7.04	1.36	4–9	7	6.91	1.3	4–9	7	7.26	1.44	5–9	7
					*p* = 0.1470 ^a^							
**Digit Span Backwards**	5.35	1.19	1–8	5	5.23	1.2	1–8	5	5.54	1.16	3–8	5.5
					*p* = 0.1552 ^a^							
**Digit span total**	12.39	2.32	5–17	12	12.14	2.23	5–17	12	12.8	2.41	8–17	13
					*p* = 0.1296 ^a^							
**STROOP**												
**Words**	113.89	15.47	76–150	113	113.91	16.35	76–150	115	113.85	14.06	85–150	113
					Difference: 0.06 CI [−5.25 5.36] *p* = 0.9830 ^b^							
**Color**	79.15	11.53	54–113	78	79.15	11.93	54–113	78	79.17	10.95	58–102	78.5
					Difference: −0.02 SD [−3.97 3.94] *p* = 0.9925 ^b^							
**Color-word**	51.04	8.58	31–71	51	51.52	9.02	31–71	50.5	50.26	7.85	33–66	51
					Difference 1.26 CI [−1.67 4.20] *p* = 0.3965 ^b^							
**Predicted color-word**	46.51	6.01	32.85–63.38	45.92	46.49	6.29	32.85–63.38	45.69	46.54	5.58	35.71–60.00	46.39
					Difference: −0.05 CI [−2.11 2.01] *p* = 0.9629 ^b^							
**Interference**	4.55	6.33	(−13.30)–(19.98)	4.54	5.06	6.2	(−11.63)–(19.98)	5.26	3.71	6.52	(−13.30)–15.22	4.2
					Difference 1.35 CI [−0.81 3.51] *p* = 0.2184 ^b^							
**Trail Making Test**												
**Test A**	21.56	7.11	10–46	20	21.42	7.22	12–46	20	21.78	6.99	10–40	21
					*p* = 0.5626 ^a^							
**Test B**	52.39	16.3	27–114	50	53.64	18.21	27–114	48	50.35	12.49	30–94	50
					*p* = 0.6604 ^a^							

Wilcoxon Test (^a^); T test (^b^); SD (standard deviation); CI (confidence interval); WMS-III (Wechsler Memory Scale-Third Edition); WAIS-III (Wechsler Adult Intelligence Scale-Third Edition); ROCF (Rey–Osterrieth Complex Figure Test).

## Data Availability

The data presented in this study are available on request from the corresponding author.
